# Biomarker studies to predict outcomes of patients with COVID-19 related acute respiratory distress syndrome measured pre and post initiation of veno venous extracorporeal membrane oxygenation

**DOI:** 10.1038/s41598-025-30047-9

**Published:** 2025-12-03

**Authors:** Mazen F. Odish, Hunter Gage, Michael T. Y. Lam, Mark Hepokoski, Travis Pollema, Khang Tong, Lin Liu, Atul Malhotra, Robert L. Owens, Angela Meier

**Affiliations:** 1 Department of Medicine, Division of Pulmonary, Critical Care, Sleep Medicine, and Physiology, UC San Diego, La Jolla, USA; 2 Department of Pediatrics, UC San Diego, La Jolla, USA; 3 VA San Diego Healthcare System, La Jolla, USA; 4 Department of Surgery, Division of Cardiovascular and Thoracic Surgery, UC San Diego, La Jolla, USA; 5 Altman Clinical and Translational Research Institute, UC San Diego, La Jolla, USA; 6 Herbert Wertheim School of Public Health and Human Longevity Science, UC San Diego, San Diego, USA; 7 Department of Anesthesiology, Division of Critical Care, UC San Diego, La Jolla, USA

**Keywords:** ARDS, ECMO, IL-10, Biomarkers, COVID, Survival to hospital discharge, Biomarkers, Diseases, Medical research, Risk factors

## Abstract

Veno-venous extracorporeal membrane oxygenation (V-V ECMO) is a resource intensive and expensive therapy increasingly used to treat a select group of patients with severe acute respiratory distress syndrome (ARDS). High levels of Interleukin-10 (IL-10) are reported to be predictive of mortality in non-COVID ARDS patients receiving ECMO. In this study we evaluated if levels of biomarkers including IL-10 can predict mortality if measured prior to ECMO initiation in patients with COVID-19 ARDS. A total of sixteen patients with COVID-related ARDS undergoing treatment with ECMO were included in the pre-ECMO biomarker analysis, while samples from a total of 22 patients with post-ECMO samples were analyzed. Nine of sixteen patients with pre-ECMO samples (56%) survived to hospital discharge. There was no difference in baseline demographics between survivors and non-survivors. In univariate analysis, survivors had no difference in IL-10 levels prior to ECMO initiation although we identified several other biomarkers (Ang 1 and TNF-alpha with logistical regression, Mitochondrial DNA and IP-10 with Kaplan–Meier analysis) that differed significantly between survivors and non-survivors. Moreover, as opposed to a prior study, area under the curve (AUC) analysis showed that IL-10 levels were not predictive of survival. We advise caution for utilizing a single biomarker as outcome predictor in this highly complex population with often long clinical courses despite our promising results.

**Supplementary Information:**

The online version contains supplementary material available at 10.1038/s41598-025-30047-9.

## Introduction

Veno-venous (V-V) extracorporeal membrane oxygenation (ECMO) is increasingly utilized to treat patients with the most severe form of acute respiratory distress syndrome (ARDS). ARDS is often, but not exclusively, a consequence of severe infection, and harbors extraordinarily high mortality^1^. Recent guidelines have adopted ECMO use for this condition^2,3^. Despite randomized controlled trials showing equipoise^4^, clinical datasets such as the ELSO database^5^ demonstrate a survival of patients placed on V-V ECMO for ARDS of approximately 50%. Despite its potential benefits, ECMO is a scarce, expensive and resource-intensive therapy, particularly for patients with prolonged ECMO and hospital courses^6^. Moreover, patient selection for V-V ECMO (who benefits?) is controversial and an area of continued research.

Biomarkers have been suggested to help stratify which patients will be most likely to survive and therefore should receive ECMO, or on the other hand, which patients are not expected to benefit and should not receive this intervention^7–9^. In ARDS, several biomarkers have been shown to be associated with mortality^10^. For patients with ARDS requiring VV-ECMO, IL-10 is one cytokine that has been proposed as a potential biomarker to predict mortality^9^. IL-10 is a key immunomodulatory cytokine that is generally considered anti-inflammatory due to its inhibitory effects on macrophages, monocytes, dendritic cells, and CD4 T cells^11–13^. Here we examined levels of biomarkers, including IL-10, prior to ECMO initiation and within 24 h post-ECMO initiation to determine if exploratory biomarkers could predict mortality in patients with severe COVID-related ARDS who undergo ECMO and therefore aid in patient selection.

## Methods

This data set was acquired as part of a prospective cohort study performed between 3/1/2020 to 11/1/2020 with the intent to study the effect of protocolized ventilator changes (driving pressure) on blood biomarkers of inflammation and lung injury^14^. The study was approved by the University of California, San Diego Human Research Protections Program Institutional Review Board (protocol #191465). Informed consent was obtained from all subjects, and all methods were carried out in accordance with relevant guidelines and regulations. The inclusion criteria, study protocol, and primary results were previously published, although the hypotheses and results of the present study are all novel^14^. In brief, patients with COVID-related severe ARDS who underwent ECMO therapy were enrolled. Following enrollment, patients received a protocolized decrease in their driving pressure to maximize lung rest and plasma biomarkers were measured at predetermined time points. These timepoints include 30 min prior to ECMO initiation, 30 min after ECMO initiation, and then three more times over the next 24 h. A detailed protocol description was previously published^14^.

Baseline demographics and ECMO biomarkers for survivors and non-survivors were summarized as mean (standard deviation) or median (interquartile range) and count (%) for continuous and categorical variables respectively and compared using Wilcoxon Rank Sum test for continuous variables and Fisher’s exact test for categorical variables.

Associations between biomarkers and Sequential Organ Failure Assessment (SOFA) score, BMI, and gender were evaluated using Spearman’s rank correlation test for continuous variables (SOFA, BMI), and Wilcoxon Rank Sum test for categorical variables (gender) separately for pre-ECMO and post-ECMO biomarkers. These metrics were chosen a priori based on prior publications^15–18^.

Receiver operating characteristic (ROC) analyses were conducted to analyze the predictive ability of ECMO biomarkers on mortality with cutoffs determined by Youden’s index. The performance metrics sensitivity, specificity, positive predictive value (PPV), negative predictive value (NPV), and the p-value from a chi-squared test on the confusion matrix (to test whether the cutoffs determined by the ROC analysis perform better than random chance at predicting the outcome) were reported for each biomarker.

Odds ratios (OR) and 95% confidence intervals (CI) were calculated for significant biomarkers dichotomized by the cutoff from ROC analysis using logistic regression with mortality as the outcome. Kaplan–Meier Curves stratified by cutoffs were plotted and compared using log-rank tests. P < 0.05 was interpreted as statistically significant.

## Results

### Demographics

Twenty-six participants were initially enrolled. One participant was found to have an intracerebral hemorrhage after ECMO initiation and was therefore withdrawn from the study. Ten patients required mobile ECMO initiation (ECMO initiated by our mobile team at a facility that does not offer ECMO and transported to our hospital), resulting in 9 of them having no pre-ECMO samples^14^. Sixteen patients who had pre-ECMO samples were included, and their baseline demographics are reported in Table 1. The etiology of ARDS was COVID-19 in this cohort. Nine out of 16 (56%) of patients with pre-ECMO samples survived to hospital discharge. There was no difference in baseline demographics between survivors and non-survivors for patients with a pre-ECMO sample (n = 16, all p’s > 0.10). Three patients had their post-ECMO sample drawn at a different time point and were therefore excluded, leaving a total of 22 post-ECMO patient samples.

**Table 1:** Baseline (pre-ECMO) characteristics (n = 16) between survivors (n = 9) and non-survivors (n = 7) with patients that had a pre-ECMO plasma sample.

	Death N = 7	Survival N = 9	Overall N = 16	p-value
Age	48.57 (7.83)	47.33 (8.47)	47.88 (7.95)	0.92
Gender				
Female	2 (28.6%)	1 (11.1%)	3 (18.8%)	0.55
Male	5 (71.4%)	8 (88.9%)	13 (81.2%)	
Race				
White	7	7	14	–
Mixed/other	0	2	2	–
Hispanic ethnicity	4	6	10	> 0.99
PMH				
CVA	0	0	0	–
HIV/AIDS	0	0	0	–
Cardiac history (CHF, CAD, PAD)	0	0	0	–
Solid cancer	1	0	1	> 0.99
Diabetes	2	2	4	–
Collagen vascular disease	0	0	0	–
Solid organ transplant	0	0	0	–
CKD	0	0	0	–
Asthma	1	1	2	–
History of smoking	0	1 (4 of unknown status)	1	–
Body mass index (kg/m^2^)	30.51 (3.39)	31.29 (4.98)	30.95 (4.24)	> 0.99
ICU SOFA	9.57 (2.64)	8.78 (2.05)	9.12 (2.28)	0.39
Intubation days prior to ECMO initiation	5.7 (2.5) 6 (4–11)	8.9 (5.5) 9 (5–11)	7.5 (4.6) 7.5 (4–9.5)	0.2
Actual tidal volume (mL)	375.29 (77.38)	392.22 (78.66)	384.81 (75.96)	0.68
Mean airway pressure (mmHg)	20.42 (3.84)	19.65 (5.29)	19.99 (4.58)	0.96
PEEP (cmH_2_O)	13.14 (2.54)	12.22 (5.12)	12.62 (4.10)	0.83
Peak inspiratory pressure (cmH_2_O)	31.57 (5.80)	30.00 (5.63)	30.69 (5.57)	0.75
FiO2 at time of ABG	*n* = 5 100.00 (0.00)	*n* = 7 87.14 (17.04)	*n* = 12 92.50 (14.22)	0.14
pH	*n* = 5 7.35 (0.08) 7.33 (7.26–7.36)	*n* = 7 7.37 (0.09) 7.33 (7.29–7.40)	*n* = 12 7.36 (0.08) 7.33 (7.28–7.38)	0.75
PaCO_2_ (mmHg)	*n* = 5 80.20 (25.36) 61 (59–81)	*n* = 7 68.00 (10.92) 64.5 (59.8–76.5)	*n* = 12 73.08 (18.40) 62 (59.5–79.5)	0.46
PaO_2_ (mmHg)	*n* = 5 73.40 (12.70) 81 (74.5–86)	*n* = 7 78.71 (16.11) 79.5 (73.5–88)	*n* = 12 76.50 (14.41) 80 (73–87)	0.64
Bicarbonate, measured (mmol/L)	n = 7 32.6 (6.9) 32 (27–38.5)	n = 9 31.7 (5.3) 32 (30–33)	n = 16 32 (5.8) 32 (29.3–37.3)	0.94
Total ECMO days	33.43 (25.32)	21.56 (16.17)	26.75 (20.81)	0.34

Data are represented as mean (SD), except for gender, which is represented as number (%). pH, PaCO_2_, PaO_2_, and Bicarbonate are represented as mean (SD) followed by median (IQR). P-values for continuous variables from Wilcoxon Rank Sum test. P-values for categorical variables from Fisher’s exact test. *CHF* Congestive heart failure, *PEEP* Positive end-expiratory pressure.

### Univariate analysis of biomarkers

**Fig. 1:**
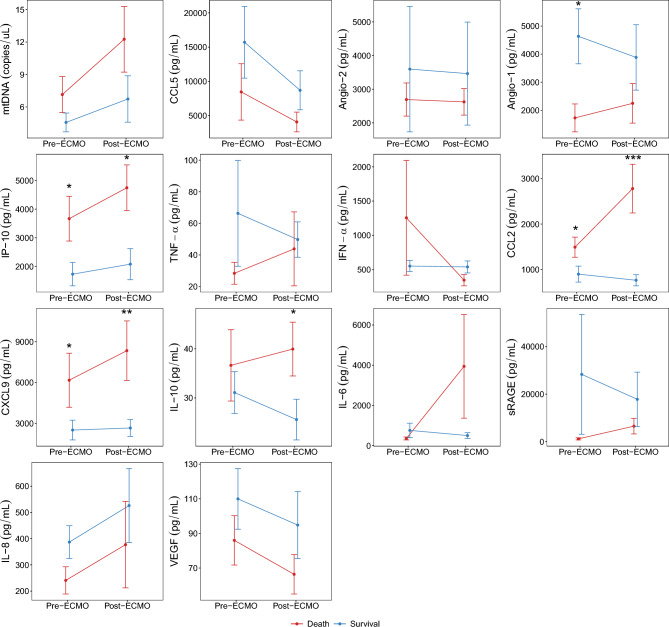
Biomarker levels measured pre-ECMO (n = 16) and within 24 h post-ECMO (n = 22) in survivors and non-survivors. Data are represented as mean ± standard error of the mean (SEM). Statistical comparisons between survivors and non-survivors were made via Wilcoxon Rank Sum tests. *p < 0.05, **p < 0.01, ***p < 0.001.

To determine whether biomarkers measured pre-ECMO or biomarkers drawn within 24 h post-ECMO initiation were correlated with outcomes, we compared levels between survivors and non-survivors. In the univariable analysis of pre-ECMO biomarkers, IL-10 was not statistically different between survivors vs. non-survivors, (mean 31.1 pg/mL survivor vs. 33.5 non-survivor, p > 0.99), see Fig. 1 and Table 2. However, the survivors had significantly lower mean pre-ECMO levels of IP-10 (p = 0.04), CCL2 (p = 0.02), CXCL9 (p = 0.04), and significantly higher mean pre-ECMO levels of Angio-1 (p = 0.04), compared to non-survivors. In the same univariable analysis, in post-ECMO samples (drawn within 24 h of ECMO initiation), the following biomarkers were statistically lower in survivors compared to non-survivors: IL-10 (p = 0.04), IP-10 (p = 0.01), CCL2 (p < 0.001), CXCL9 (p = 0.004). Markers that trended lower but were not statistically significant included TNF-α (p = 0.06) and CCL5 (p = 0.08), and borderline significantly higher for IL-8 (p = 0.05) (Fig. 1 and Supplemental Table S1). Taken together, in a univariable analysis of pre-ECMO samples, IP-10, CCL2, CXCL9 and Angio-1 were predictors of mortality, but IL-10 was not. In post-ECMO samples, IL-10, IP-10, CCL2 and CXCL9 were predictors of mortality.

**Table 2:** Pre-ECMO biomarkers by survivors (n = 9) vs. non-survivors (n = 7).

	Death	Survival	Overall		p-value
	(*n* = 7)	(*n* = 9)	(*n* = 16)		
mtDNA	7.14 (4.41)	4.57 (2.61)	5.70 (3.63)		0.47
CCL5	8449.6 (10,901.5)	15,706.2 (15,734.7)	12,531.5 (13,906.9)		0.21
Angio-2	2692.7 (1304.6)	3597.6 (5592.1)	3201.7 (4192.1)		0.41
Angio-1	1730.1 (1314.3)	4634.7 (2939.5)	3363.9 (2741.2)		0.04
IP-10	3667.0 (2063.6)	1729.3 (1227.9)	2577.0 (1869.0)		0.04
TNF α	28.4 (18.4)	66.3 (100.4)	49.7 (76.8)		0.17
IFN α	1255.6 (2214.2)	551.9 (242.7)	859.7 (1456.8)		0.41
CCL2	1491.1 (587.2)	897.8 (527.8)	1157.4 (615.6)		0.02
CXCL9	6171.3 (5252.2)	2509.6 (2178.2)	4111.6 (4133.3)		0.04
IL-10	36.6 (19.3)	31.1 (12.8)	33.5 (15.6)		> 0.99
IL-6	355.8 (192.4)	757.9 (1077.2)	582.0 (822.2)		0.92
sRAGE	1156.9 (1202.7)	28,310.0 (75,619.8)	16,430.5 (56,955.3)		0.09
IL-8	240.8 (137.2)	386.9 (187.6)	323.0 (178.6)		0.14
VEGF	86.0 (37.7)	110.0 (52.5)	99.5 (46.8)		0.41

mtDNA units in copies/uL. Other biomarker units in pg/mL. Data are represented as mean (SD). P-values from Wilcoxon Rank Sum test (n = 16). mtDNA, mitochondrial DNA. CCL5, C–C Motif Chemokine Ligand 5. Angio-1, angiotensin-1. Angio-2, angiotensin-2. IP-10, Interferon-γ-induced protein 10. TNF-α, tumor necrosis factor-α. INF-α, interferon-α. CCL2, chemokine (C–C motif) ligand 2. CXCL9, C-X-C motif chemokine ligand 9. IL-10, interleukin-10. IL-6, interleukin-6. sRAGE, soluble receptor for advanced glycation end-products. *IL-8* Interleukin-8, *VEGF* Vascular endothelial growth factor.

### ROC analysis

For pre-ECMO biomarkers, significant AUCs were found for mitochondrial DNA (mtDNA) (p = 0.049), CCL5 (p = 0.049), Angio-1 (p = 0.006), IP-10 (p = 0.01), TNF-α (p = 0.01), CCL2 (p = 0.006), CXCL9 (p = 0.02), sRAGE (p = 0.049), and IL-8 (p = 0.049). For these biomarkers, the AUC, sensitivity, specificity, positive and negative predictive values, and p-values are presented in Supplemental Table S2 and Supplemental Fig. S1. The optimal IL-10 cutoff pre-ECMO was determined to be 23.2 with AUC of 0.51 (p = 0.18), indicating that IL-10 levels pre-ECMO initiation in our studied cohort did not differentiate between survivors and non-survivors (Supplemental Table S2 and Supplemental Fig. S1).

In the post-ECMO samples, IL-10 had a statistically significant AUC (cutoff: ≥ 24.8, AUC: 0.76, p = 0.01), and higher IL-10 was associated with higher likelihood of death from the univariate logistic regression (OR = 11.7; 95% CI 1.77, 115.8; p = 0.02, Table 3). Other post-ECMO biomarkers that were found to have a significant AUC were mtDNA (p = 0.045), CCL5 (p = 0.03), IP-10 (p = 0.01), TNF-α (p = 0.009), INF-α (p = 0.03), CCL2 (p < 0.001), CXCL9 (p = 0.011), IL-6 (p = 0.045), and IL-8 (p = 0.003) (Supplemental Table S2and Supplemental Fig. S1).

**Table 3:** Univariable logistic regression analyses of association between mortality and biomarkers measured pre-ECMO. Odds ratios > 1 indicate a higher risk of mortality.

	Univariable analysis		
	Unadjusted odds ratio	95% CI	p-value
mtDNA (≥ 7.75 copies/uL)	10*.*667	(1*.*056, 262*.*560)	0*.*070
CCL5 (≤ 4340 pg/mL)	8*.*750	(1*.*048, 112*.*539)	0*.*061
Angio-1 (≤ 3800 pg/mL)	27*.*857	(2*.*235, 4042*.*219)	0*.*007**
IP-10 (≥ 2620 pg/mL)	10*.*667	(1*.*056, 262*.*560)	0*.*070
TNF α (≤ 26.9 pg/mL)	21*.*000	(2*.*038, 558*.*550)	0*.*024*
CCL2 (≥ 832 pg/mL)	12*.*000	(1*.*256, 290*.*575)	0*.*054
CXCL9 (≥ 1680 pg/mL)	7*.*500	(0*.*799, 175*.*661)	0*.*113
sRAGE (≤ 945 pg/mL)	10*.*667	(1*.*056, 262*.*560)	0*.*070
IL-8 (≤ 189 pg/mL)	6*.*000	(0*.*561, 145*.*572)	0*.*170

### Kaplan–Meier curves

**Fig. 2:**
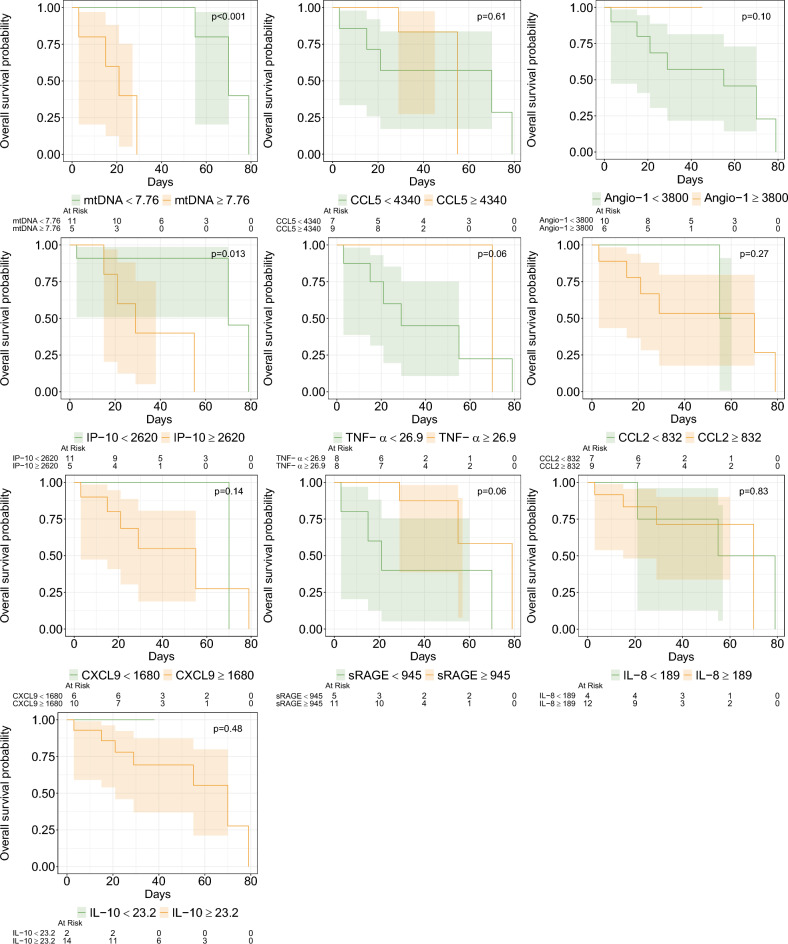
Kaplan–Meier curves stratified by pre-ECMO biomarker level (with cutoffs having significant AUC determined by ROC analysis) with survival to hospital discharge as the outcome. Statistical comparison was assessed via log-rank tests.

Significant improvement in survival was found in patients with pre-ECMO mtDNA < 7.76 (p < 0.001) or IP-10 ≤ 2620 ng/ml (p = 0.01). Post-ECMO patients with a mtDNA < 10.3 (p = 0.001), or TNF-α ≥ 14.2 ng/ml (p = 0.01), or CCL2 ≤ 1540 ng/ml (p = 0.004) had improved survival (Supplemental Fig. S2). The Kaplan–Meier curve for pre-ECMO IL-10 (cutoff of 23.2 ng/ml) resulted in a non-significant difference in survival (p = 0.2), see Fig. 2.

In summary, our Kaplan–Meier analysis found that pre-ECMO: mtDNA, IP-10 and post-ECMO: mtDNA, TNF-α, and CCL2 were associated with differences in survival.

### Biomarker association analysis

Pre and post-ECMO biomarkers were then correlated with SOFA score, BMI, and gender. These parameters were chosen a priori based on prior publications indicating a possible association with these metrics and biomarkers.

There was no significant correlation between pre-ECMO IL-10 and SOFA (spearman rho = 0.22, p = 0.40) or BMI (rho = -0.48, p = 0.06), see Supplementary Table S4 for other biomarkers. For other pre-ECMO biomarkers, higher IFN-α was significantly correlated with lower SOFA (rho = -0.57, p = 0.02), and higher IL-8 was significantly correlated with lower BMI (rho = -0.59, p = 0.02)**.**

Similarly, there was no significant correlation between post-ECMO IL-10 and SOFA (p = 0.14) or BMI (p = 0.14). For other post-ECMO biomarkers, CCL5 and IFN-α were both negatively correlated with SOFA (p = 0.002 and p = 0.003, respectively)**.** Interestingly, there was no significant difference found in either pre- or post-ECMO biomarkers between male and female (see Supplementary Table S5).

## Discussion

While ECMO can be a lifesaving therapy, it is only available for a very limited number of patients through specialized centers. The question of who benefits from ECMO is still debated and investigated. Biomarkers such as IL-10 have been proposed to be associated with outcomes on ECMO even if measured prior to ECMO initiation. As opposed to a previously published investigation by *Liu *et al.^9^, in our study of COVID-19 ARDS patients undergoing ECMO treatment, we did not find that IL-10 predicted outcomes. Importantly, the levels of IL-10 in our study were generally lower than those reported by *Liu *et al.: Patients in their study had an average IL-10 level of approximately 75 pg/mL in survivors and 500 pg/mL in non-survivors, and an optimal cutoff value of 88.9 pg/mL was used for their ROC analysis. In our study, the average IL-10 level pre-ECMO was 31.09 pg/mL in survivors and 36.64 pg/mL in non-survivors, which is significantly lower than in *Liu *et al*.*’s cohort. Of note, *Liu *et al*.*’s study was conducted prior to the COVID-19 pandemic, while our cohort consisted exclusively of COVID-19 patients. Studies of patients with non-COVID-19 viral pneumonia have found median IL-10 levels of approximately 1–5 pg/mL^19–21^, which is lower than those reported by *Liu *et al*.*, suggesting that the differences in IL-10 levels between our studies were not due to COVID-19 versus non-COVID ARDS. Another key difference between our study and *Liu *et al. is that their study included patients with diverse etiologies of ARDS (viral pneumonia, bacterial pneumonia, trauma, aspiration pneumonitis, extrapulmonary sepsis, autoimmune diseases, and post-operative), while ours focused solely on COVID-19 related ARDS. Thus, the predictive power of IL-10 levels for ECMO outcomes might differ depending on the specific cause of ARDS and should not be generalized to all ARDS patients.

There were a few exploratory biomarkers that, interestingly had statistically significant associations with mortality. In our univariate logistic regression, lower levels of pre-ECMO Angio-1 and TNF-ɑ were correlated with increased mortality. Angiopoietin- 1 (Angio-1), a ligand for the endothelial Tie2 receptor, has been shown to be able to inhibit leukocyte- endothelium interactions and to enhance endothelial survival and stabilize vasculature^22^. Increased levels are therefore beneficial in ARDS and indeed, our data show that lower levels of Angio-1 were associated with increased mortality. TNF levels have been shown to be increased in ARDS^23^- in our study lower levels were correlated with mortality when measured prior to ECMO initiation.

Higher levels of pre-ECMO mtDNA and IP-10 were associated with a decreased time to death in our Kaplan Meier analysis. It is known that inflammatory markers correlate with disease severity and outcomes in ARDS and are utilized in subclassifying this heterogenous disease^24^. Specifically, mtDNA is a damage-associated molecular pattern (DAMP) that can trigger inflammation by activating TLR9, NF-kB, and the NLRP3 inflammasome^25^. Elevated plasma mtDNA levels are correlated with increased mortality in medical ICU patients^26^. The SARS-CoV-2 envelope protein can induce apoptosis and mtDNA release, leading to elevated mtDNA levels in COVID-19 patients^27^. In our study, high levels of mtDNA were associated with decreased time to death when measured before ECMO initiation. IP-10, a chemokine that is secreted in response to Interferon Gamma, has been shown to predict risk of progression to severe respiratory failure or death from COVID-19^28^. When measured prior to or post ECMO initiation, IP-10 was also associated with decreased time to death in our study.

There are several limitations to our study: First, the cohort size was small (16 pre-ECMO samples and 22 post-ECMO samples), which could have resulted in high variance and model overfitting for ROC curves, and did not allow for multivariate analysis. Second, our study was conducted at a single site. Third, the patient population was majority single race and only included patients with COVID-19 related ARDS, making the population relatively homogenous. Therefore, additional research is needed in order to determine whether these exploratory, positively associated biomarkers indeed can predict outcomes on a larger scale, particularly in patients with more diverse causes of ARDS. Finally, differences in biomarker processing, storage, and assays may compromise reproducibility across centers.

In summary, we suggest caution when considering a single biomarker as a predictive marker for ARDS outcomes on ECMO. Overall, many patients on ECMO for ARDS have prolonged and complicated clinical courses, with many etiologies of respiratory failure, thus, finding one single biomarker that will accurately predict outcomes in this entire population is unlikely. Nonetheless, our study, with the limitations stated above, suggests other exploratory biomarkers as possible candidates, a combination of which could be considered in future studies aiming at predicting mortality before ECMO initiation.

## Supplementary Information

Supplementary Information.

## Data Availability

Data collected will be made available on reasonable request from the Corresponding author and approval of the authors.
