# Supplementary material for: Biomarker studies to predict outcomes of patients with COVID-19 related acute respiratory distress syndrome measured pre and post initiation of veno venous extracorporeal membrane oxygenation
Source: Sci Rep. 2025 Dec 3;16:511. doi: 10.1038/s41598-025-30047-9 (PMC12775480; doi:10.1038/s41598-025-30047-9)
Supplement: Supplementary file 1 — Supplementary Information. [file 41598_2025_30047_MOESM1_ESM.docx]

# **Supplementary Appendix**

Biomarker studies to predict outcomes of patients with COVID-19 related acute respiratory distress syndrome measured pre and post initiation of veno venous extracorporeal membrane oxygenation

**Authors**: Mazen Odish, MD^1*^; Hunter Gage^2*^, Michael Lam, MD^1,3^; Mark Hepokoski, MD^1,3^; Travis Pollema, DO^3,4^; Khang Tong, MS^5^; Lin Liu, PhD^6^; Atul Malhotra, MD^1^; Robert L. Owens, MD^1^; Angela Meier, MD, PhD^7^

**Institutions and Affiliations:**

^*^Co-first Authors

^1^UC San Diego Department of Medicine, Division of Pulmonary, Critical Care, Sleep Medicine, and Physiology

^2^UC San Diego Division of Pediatrics

^3^Veteran’s Affairs, San Diego Medical Center

^4^UC San Diego Department of Surgery, Division of Cardiovascular and Thoracic Surgery

^5^UC San Diego, Altman Clinical and Translational Research Institute

^6^UC San Diego, Herbert Wertheim School of Public Health and Human Longevity Science

^7^UC San Diego Department of Anesthesiology, Division of Critical Care

|  | Death | Survival | Overall | p-value | |
| --- | --- | --- | --- | --- | --- |
|  | (*n* = 12) | (*n* = 10) | (n = 22) |  | |
| mtDNA | 12.3 (10.5) | 6.78 (6.8) | 9.8 (9.3) | | 0.18 |
| CCL5 | 4056.9 (4998.4) | 8682.1 (9011.2) | 6159.3 (7310.5) | | 0.08 |
| Angio-2 | 2624.0 (1365.1) | 3464.6 (4839.0) | 3006.1 (3345.9) | | 0.50 |
| Angio-1 | 2248.1 (2434.1) | 3883.0 (3684.4) | 2991.2 (3100.9) | | 0.25 |
| IP-10 | 4746.0 (2766.0) | 2078.5 (1714.2) | 3533.5 (2667.4) | | 0.01 |
| TNF$\text{α}$ | 43.9 (81.1) | 49.8 (35.4) | 46.5 (63.2) | | 0.06 |
| IFN$\text{α}$ | 346.1 (289.5) | 539.1 (276.5) | 433.8 (293.9) | | 0.14 |
| CCL2 | 2780.0 (1855.67) | 763.7 (393.0) | 1863.5 (1710.5) | | <0.001 |
| CXCL9 | 8343.3 (7579.9) | 2660.3 (1965.7) | 5760.1 (6335.6) | | 0.004 |
| IL-10 | 40.0 (19.0) | 25.6 (13.1) | 33.4 (17.8) | | 0.04 |
| IL-6 | 3943.1 (8927.3) | 500.6 (456.8) | 2378.4 (6701.7) | | 0.20 |
| sRAGE | 6506.4 (11348.7) | 17809.2 (36230.5) | 11644.0 (25752.9) | | 0.62 |
| IL-8 | 377.1 (571.1) | 526.2 (446.1) | 444.9 (511.8) | | 0.0503 |
| VEGF | 66.4 (39.4) | 94.8 (61.1) | 79.3 (51.2) | | 0.46 |

**Supplementary Table S1:** Biomarkers at Post-ECMO (n=22) by survivors vs. non-survivors. mtDNA units in copies/uL. Other biomarker units in pg/mL. Data are represented as mean (SD). P-values from Wilcoxon Rank Sum test. mtDNA, mitochondrial DNA. CCL5, C-C Motif Chemokine Ligand 5. Angio-1, angiotensin-1. Angio-2, angiotensin-2. IP-10, Interferon-γ-induced protein 10. TNF-α, tumor necrosis factor-α. INF-α, interferon-α. CCL2, chemokine (C-C motif) ligand 2. CXCL9, C-X-C motif chemokine ligand 9. IL-10, interleukin-10. IL-6, interleukin-6. sRAGE, soluble receptor for advanced glycation end-products. IL-8, interleukin-8. VEGF, vascular endothelial growth factor.

|  | **Pre-ECMO** | | | | | | | | **Post-ECMO** | | | | | | | |
| --- | --- | --- | --- | --- | --- | --- | --- | --- | --- | --- | --- | --- | --- | --- | --- | --- |
|  | AUC | Direction | Cutoff Value  (pg/mL) | Sensitivity % | Specificity % | PPV % | NPV % | p-value | AUC | Direction | Cutoff Value (pg/mL) | Sensitivity % | Specificity % | PPV % | NPV % | p-value |
| mtDNA | 0.62 | *≥* | 7.76 | 57.1 | 88.9 | 80.0 | 72.7 | 0.049 | 0.68 | *≥* | 10.3 | 50.0 | 90.0 | 85.7 | 60.0 | 0.045 |
| CCL5 | 0.70 | *≤* | 4340 | 71.4 | 77.8 | 71.4 | 77.8 | 0.049 | 0.73 | *≤* | 5810 | 91.7 | 50.0 | 68.8 | 83.3 | 0.03 |
| Angio-2 | 0.64 | *≥* | 2810 | 42.9 | 88.9 | 75.0 | 66.7 | 0.15 | 0.59 | *≥* | 1610 | 83.3 | 50.0 | 66.7 | 71.4 | 0.10 |
| Angio-1 | 0.81 | *≤* | 3800 | 100.0 | 66.7 | 70.0 | 100.0 | 0.006 | 0.65 | *≤* | 4310 | 91.7 | 40.0 | 64.7 | 80.0 | 0.08 |
| IP-10 | 0.81 | *≥* | 2620 | 71.4 | 88.9 | 83.3 | 80.0 | 0.01 | 0.81 | *≥* | 2990 | 75.0 | 80.0 | 81.8 | 72.7 | 0.01 |
| TNF$\text{α}$ | 0.71 | *≤* | 26.9 | 85.7 | 77.8 | 75.0 | 87.5 | 0.01 | 0.74 | *≤* | 14.2 | 50.0 | 100.0 | 100.0 | 62.5 | 0.009 |
| IFN$\text{α}$ | 0.64 | *≤* | 596 | 85.7 | 55.6 | 60.0 | 83.3 | 0.09 | 0.69 | *≤* | 325 | 66.7 | 80.0 | 80.0 | 66.7 | 0.03 |
| CCL2 | 0.84 | *≥* | 832 | 100.0 | 66.7 | 70.0 | 100.0 | 0.006 | 0.94 | *≥* | 1540 | 83.3 | 100.0 | 100.0 | 83.3 | <0.001 |
| CXCL9 | 0.81 | *≥* | 1680 | 100.0 | 55.6 | 63.6 | 100.0 | 0.02 | 0.85 | *≥* | 3610 | 83.3 | 70.0 | 76.9 | 77.8 | 0.01 |
| IL-10 | 0.51 | *≥* | 23.2 | 100.0 | 22.2 | 50.0 | 100.0 | 0.18 | 0.76 | *≥* | 24.8 | 83.3 | 70.0 | 76.9 | 77.8 | 0.01 |
| IL-6 | 0.48 | *≥* | 155 | 100.0 | 22.2 | 50.0 | 100.0 | 0.18 | 0.67 | *≥* | 757 | 50.0 | 90.0 | 85.7 | 60.0 | 0.045 |
| sRAGE | 0.76 | *≤* | 945 | 57.1 | 88.9 | 80.0 | 72.7 | 0.049 | 0.57 | *≤* | 502 | 25.0 | 100.0 | 100.0 | 52.6 | 0.09 |
| IL-8 | 0.73 | *≤* | 189 | 57.1 | 88.9 | 80.0 | 72.7 | 0.049 | 0.75 | *≤* | 194 | 58.3 | 100.0 | 100.0 | 66.7 | 0.003 |
| VEGF | 0.64 | *≤* | 126 | 100.0 | 33.3 | 53.8 | 100.0 | 0.09 | 0.60 | *≤* | 62 | 58.3 | 70.0 | 70.0 | 58.3 | 0.18 |

**Supplementary Table S2**: Summary results of ROC analysis for pre-ECMO and post-ECMO plasma samples. The direction is the predicting cutoff for mortality. Optimal cutoff values were determined by Youden’s index.

|  | **Univariable Analysis** | | |
| --- | --- | --- | --- |
|  | Unadjusted Odds Ratio | 95% CI | p-value |
| mtDNA (≥10.3 copies/uL) | 6*.*4 | (0*.*79, 138*.*9) | 0*.*12 |
| CCL5 (≤5810 pg/mL) | 5*.*0 | (0*.*78, 44*.*9) | 0*.*11 |
| IP-10 (≥2990 pg/mL) | 12*.*0 | (1*.*84, 118*.*7) | 0*.*02 |
| TNF$\text{α}$ (≤14.2 pg/mL) | 21*.*0 | (1*.*93, 2915) | 0*.*009 |
| IFN$\text{α}$ (≤325 pg/mL) | 8*.*0 | (1*.*29, 73*.*1) | 0*.*04 |
| CCL2 (≥1540 pg/mL) | 57*.*0 | (5*.*0, 8102) | *<*0*.*001 |
| CXCL9 (≥3610 pg/mL) | 11*.*7 | (1*.*77, 115*.*8) | 0*.*02 |
| IL-10 (≥24.8 pg/mL) | 11*.*7 | (1*.*77, 115*.*8) | 0*.*02 |
| IL-6 (≥757 pg/mL) | 6*.*4 | (0*.*79, 138*.*9) | 0*.*12 |
| IL-8 (≤194 pg/mL) | 21*.*0 | (1*.*93, 2915) | 0*.*009 |

**Supplementary Table S3:** Logistic regression results for significant demographic characteristics and biomarkers from ROC analysis in the post-ECMO period.

|  | **Pre-ECMO** | | | | **Post-ECMO** | | | |
| --- | --- | --- | --- | --- | --- | --- | --- | --- |
|  | SOFA | p-value | BMI | p-value | SOFA | p-value | BMI | p-value |
| mtDNA | 0.02 | 0.93 | -0.40 | 0.13 | 0.05 | 0.82 | -0.42 | 0.05 |
| CCL5 | -0.26 | 0.34 | -0.28 | 0.29 | -0.61 | 0.002 | -0.12 | 0.61 |
| Angio-2 | -0.30 | 0.28 | 0.26 | 0.34 | -0.25 | 0.26 | 0.01 | 0.98 |
| Angio-1 | -0.13 | 0.64 | -0.41 | 0.11 | -0.20 | 0.36 | -0.11 | 0.62 |
| IP-10 | -0.08 | 0.78 | 0.32 | 0.23 | -0.16 | 0.49 | 0.1 | 0.67 |
| TNF$\text{α}$ | 0.03 | 0.92 | -0.37 | 0.17 | -0.34 | 0.12 | -0.36 | 0.11 |
| IFN$\text{α}$ | -0.57 | 0.02 | -0.27 | 0.31 | -0.61 | 0.003 | -0.24 | 0.28 |
| CCL2 | -0.03 | 0.91 | -0.25 | 0.34 | 0.04 | 0.85 | -0.16 | 0.47 |
| CXCL9 | 0.03 | 0.90 | -0.22 | 0.42 | -0.19 | 0.41 | 0.02 | 0.95 |
| IL-10 | 0.22 | 0.40 | -0.48 | 0.06 | 0.33 | 0.14 | -0.39 | 0.14 |
| IL-6 | -0.03 | 0.90 | -0.11 | 0.70 | 0.09 | 0.68 | 0.001 | 1.00 |
| sRAGE | -0.28 | 0.30 | 0.20 | 0.46 | -0.16 | 0.49 | -0.14 | 0.54 |
| IL-8 | -0.04 | 0.88 | -0.59 | 0.02 | -0.28 | 0.20 | -0.25 | 0.25 |
| VEGF | -0.01 | 0.98 | -0.48 | 0.06 | 0.09 | 0.70 | -0.22 | 0.33 |

**Supplementary Table S4**: Spearman correlation coefficients between biomarkers and ICU SOFA and BMI within 24 hours pre-ECMO and post-ECMO. Values were rounded to the nearest hundredth, except for p-values < 0.01.

|  | **Pre-ECMO** | | | | **Post-ECMO** | | | |
| --- | --- | --- | --- | --- | --- | --- | --- | --- |
|  | Female  (*n* = 3) | Male  (*n* = 13) | Overall  (*n* = 16) | p-value | Female  (*n* = 4) | Male  (*n* = 18) | Overall  (*n* = 22) | p-value |
| mtDNA | 7.68  (0.79) | 5.24  (3.89) | 5.70  (3.63) | 0.24 | 8.51 (10.7) | 10.0  (9.22) | 9.75  (9.25) | 0.48 |
| CCL5 | 20352.8  (14241.3) | 10726.5 (13752.4) | 12531.5 (13906.9) | 0.24 | 11639.9 (11001.4) | 4941.3  (6001.9) | 6159.3 (7310.5) | 0.30 |
| Angio-2 | 7217.5  (9676.3) | 2275.0 (1183.3) | 3201.7 (4192.1) | 0.90 | 5502.4 (7762.9) | 2451.4  (1184.2) | 3006.1 (3345.9) | 0.97 |
| Angio-1 | 3543.1  (3059.3) | 3322.6 (2796.9) | 3363.9 (2741.2) | 0.80 | 2010.4 (2409.7) | 3209.2  (3252.2) | 2991.2 (3100.9) | 0.34 |
| IP-10 | 4357.3  (2424.9) | 2166.2 (1552.7) | 2577.0 (1869.0) | 0.08 | 3733.1 (2684.7) | 3489.1  (2739.7) | 3533.5 (2667.4) | 0.90 |
| TNF$\text{α}$ | 23.1  (5.2) | 55.9  (84.5) | 49.7  (76.8) | 0.44 | 37.0  (21.3) | 48.7  (69.5) | 46.5  (63.2) | 0.42 |
| IFN$\text{α}$ | 500.0  (173.0) | 942.8  (1615.0) | 859.7 (1456.8) | 0.80 | 519.8 (248.8) | 414.7  (306.0) | 433.8 (293.9) | 0.39 |
| CCL2 | 1604.3  (936.3) | 1054.2  (515.8) | 1157.4 (615.6) | 0.36 | 1265.6 (951.5) | 1996.3  (1830.8) | 1863.5 (1710.5) | 0.77 |
| CXCL9 | 5576.0  (2579.4) | 3773.7 (4425.7) | 4111.6 (4133.3) | 0.08 | 4292.6 (3274.1) | 6086.2  (6861.0) | 5760.1 (6335.6) | 0.84 |
| IL-10 | 33.7  (10.0) | 33.5  (17.0) | 33.5  (15.6) | 0.70 | 31.1  (6.13) | 33.9  (19.6) | 33.4  (17.8) | 0.65 |
| IL-6 | 393.7  (220.8) | 625.5  (908.9) | 582.0  (822.2) | 0.90 | 607.6 (25.7) | 2771.9  (7387.8) | 2378.4 (6701.7) | 0.77 |
| sRAGE | 77564.2 (131832.3) | 2322.7 (2882.0) | 16430.5 (56955.3) | 0.55 | 36752.9 (54020.4) | 6064.3 (11089.4) | 11644.0 (25752.9) | 0.87 |
| IL-8 | 266.5  (67.1) | 336.0  (195.3) | 323.0  (178.6) | 0.70 | 369.4 (98.3) | 461.6  (565.9) | 444.9 (511.8) | 0.34 |
| VEGF | 89.3  (56.4) | 101.8  (46.6) | 99.5  (46.8) | 0.90 | 65.7  (30.5) | 82.4  (55.0) | 79.3  (51.2) | 0.84 |

**Supplementary Table S5**: Wilcoxon rank-sum tests between biomarkers and gender at Pre-ECMO and Post-ECMO.


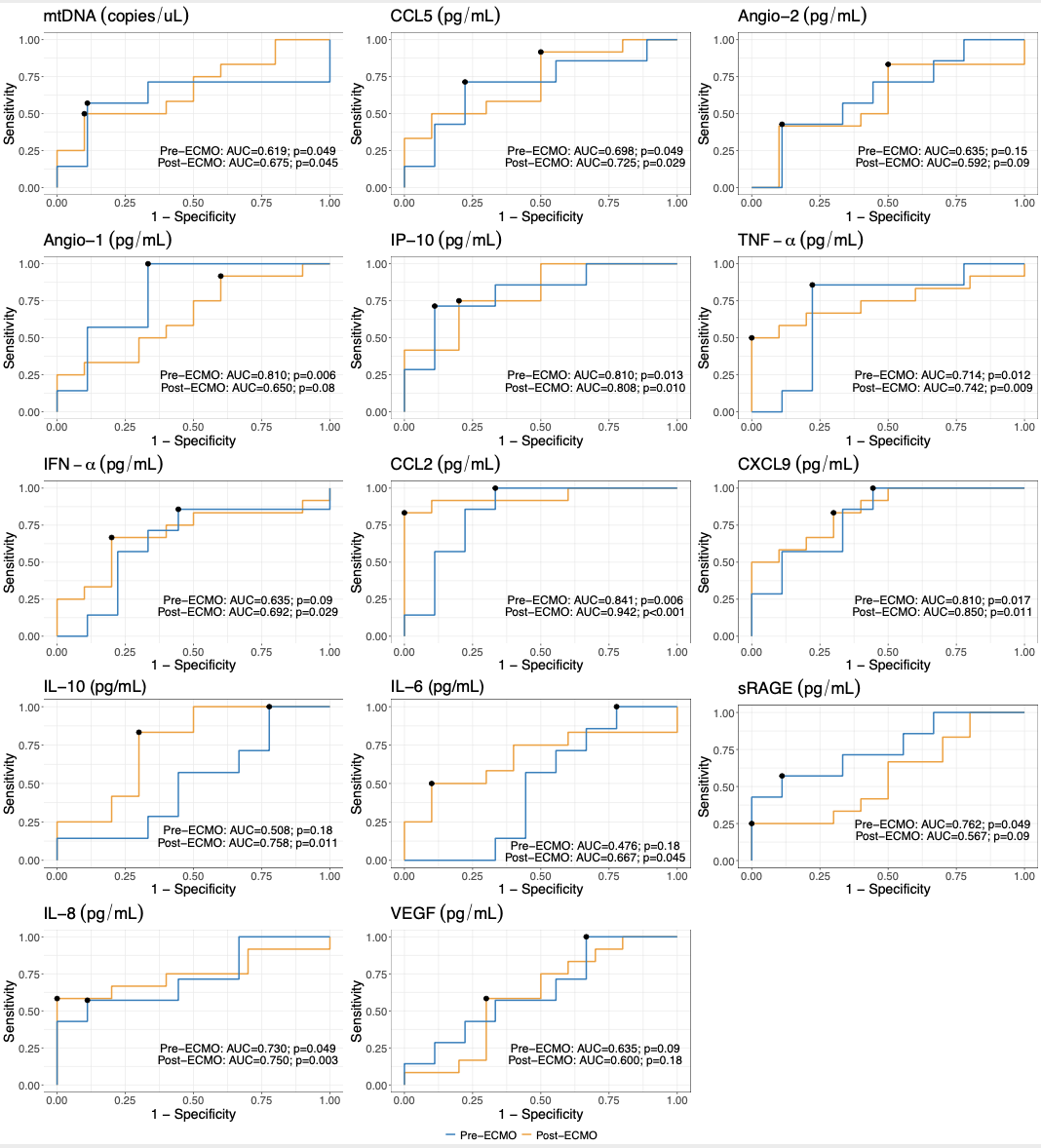


**Supplementary Figure S1:** Receiver operating characteristic (ROC) curve for biomarkers and mortality when measured pre-ECMO and within 24 hours post-ECMO. P-value from a chi-squared test on the confusion matrix to test whether the cutoffs determined by the ROC analysis perform better than random chance at predicting the outcome. Black dot indicates optimal cutoff determined by Youden’s index.


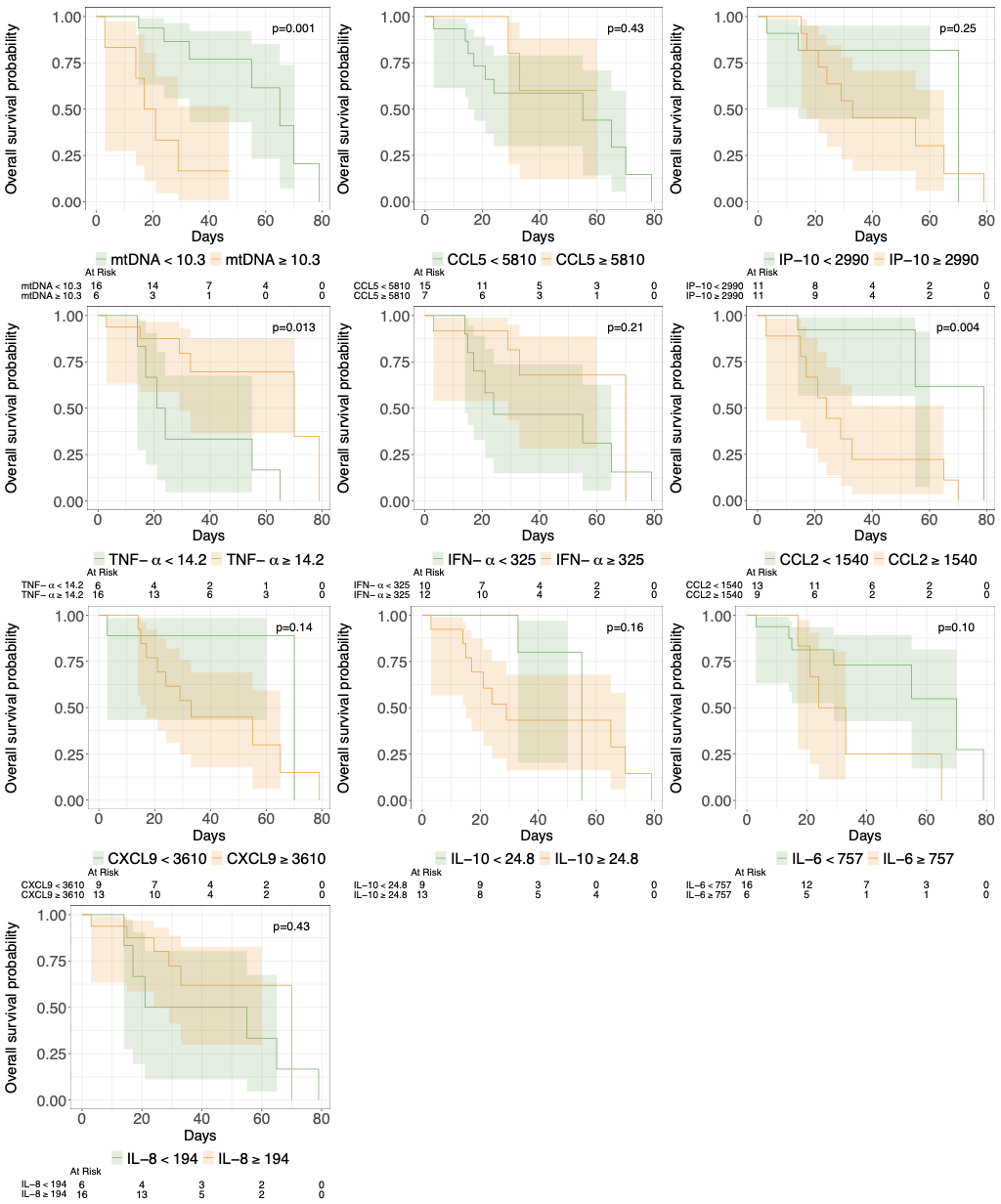


**Supplementary Figure S2**: Kaplan-Meier curves stratified by post-ECMO biomarker level with survival to hospital discharge as the outcome. Statistical comparison was assessed via log-rank tests.
